# A cure for the plague of parameters: constraining models of complex population dynamics with allometries

**DOI:** 10.1098/rspb.2013.1901

**Published:** 2013-11-07

**Authors:** Lawrence N. Hudson, Daniel C. Reuman

**Affiliations:** Imperial College London, Silwood Park, Buckhurst Road, Ascot, Berkshire SL5 7PY, UK

**Keywords:** body size, food webs, metabolic theory, predator–prey, trophic interactions

## Abstract

A major goal of ecology is to discover how dynamics and structure of multi-trophic ecological communities are related. This is difficult, because whole-community data are limited and typically comprise only a snapshot of a community instead of a time series of dynamics, and mathematical models of complex system dynamics have a large number of unmeasured parameters and therefore have been only tenuously related to real systems. These are related problems, because long time-series, if they were commonly available, would enable inference of parameters. The resulting ‘plague of parameters’ means most studies of multi-species population dynamics have been very theoretical. Dynamical models parametrized using physiological allometries may offer a partial cure for the plague of parameters, and these models are increasingly used in theoretical studies. However, physiological allometries cannot determine all parameters, and the models have also rarely been directly tested against data. We confronted a model of community dynamics with data from a lake community. Many important empirical patterns were reproducible as outcomes of dynamics, and were not reproducible when parameters did not follow physiological allometries. Results validate the usefulness, when parameters follow physiological allometries, of classic differential-equation models for understanding whole-community dynamics and the structure–dynamics relationship.

## 1. Introduction

It is important theoretically and possibly for future practical application to understand the population dynamics of species in complex ecosystems and how dynamics may depend on and also affect community structure. Dynamical features of communities, such as their stability or instability to perturbations, must certainly be related to community structure [1,2]. For instance, one interpretation of Robert May's seminal work using randomly parametrized community matrices [3–5] is that unstructured (i.e. randomly structured) complex communities are extremely unlikely to be stable. Using the same modelling approach as May, recent work showed that community matrices can imply stability if they are appropriately structured, with tightly coupled predator–prey pairs [6]. Community dynamics must, in turn, influence community structure, because structure is the outcome of dynamics up to the time of observation. For instance, species average population densities in communities tend to be approximately proportional to a power of species body mass [7–9]; power-law exponents vary substantially among ecosystems of different types but much less among ecosystems of the same type [9,10]. Exponents characterize community structure by quantifying the balance of population abundance among large- and small-bodied species that results from their trophic dynamics.

Although there clearly is a relationship between structure and dynamics, it has been difficult to study it in detail because of insufficient data and difficulties in parametrizing models. The difficulties arise because dynamical models of complex systems can have hundreds of unmeasured parameters. Parametrizing a complex model has been likened to finding a needle in a haystack: the needle represents good parameters, which cause the model to produce realistic outputs, and the haystack represents a very large space of possible parameters for the model. Evaluation of a model depends on finding good parameters, if they exist. Thus, it is hard to determine whether a model is appropriate. Worse, several competing models may be available, representing competing hypotheses about what biological and environmental factors are important. To select among hypotheses, one must find best parameters for each framework, thereby not only finding a needle in a haystack, but also finding out which of several haystacks, if any, contain needles at all. This ‘plague of parameters’ [11] pertains to all standard dynamical modelling frameworks for complex communities, including differential equation [11], community matrix [12], autoregressive [13] and Markov chain approaches [14,15].

If an abundance of population time-series data were available, then parameter values could, in principle, be inferred statistically; however, whole-community data are limited and typically provide only a snapshot or time-averaged community description instead of long time-series of species' population densities. So time-series fitting methods cannot realistically be used to infer the values of all parameters except in cases where truly exceptional data exist—usually for monotrophic communities of sessile species [14–19]. The result is that most studies of the dynamics of complex, multi-trophic communities have been very theoretical; it is not known to what extent the models and parameters used actually parallel the dynamical behaviour of real communities and provide insights about them.

Dynamical models parametrized using physiological allometries may offer a partial cure for the plague of parameters. Most parameters in some community models represent biologically interpretable quantities, such as species' maximum ingestion and respiration rates. These rates are linked to the physiology of the species and are strongly correlated with body size. Metastudy information has been used to determine the interspecific relationship between rates and body size, and model parameters for species in a community of interest can then be estimated [11]. Recent models of complex systems have used this technique to remarkably constrain the space of possible parameters [20–23]. However, not all parameters can be determined from physiological allometries. A much smaller, but still large space remains to be searched. Models parametrized with physiological allometries, despite their increasing use, have also virtually never been directly tested, i.e. it is largely unexamined whether the reduced parameter space contains parameter values that lead to ecologically sensible model behaviour (but see [24], also discussed below). Such validation efforts are important, and increasingly so, as a growing amount of research is based on insights gained from these models that are, in fact, ecologically irrelevant if the models do not accurately represent ecosystems.

We confronted physiological–allometry–parametrized differential equation models with one of the most highly resolved and complete multi-trophic community datasets available, from Tuesday Lake, MI, USA. We examined whether there are sets of parameters, in the reduced space that remains after physiological parametrization, that cause the model to reproduce the most important structural patterns that have been observed in real communities. Specifically, we asked whether the model could be made to reproduce: (i) average species population densities and how these vary with species body size both within and among trophic and taxonomic groupings; (ii) abundance–spectrum representations of population densities in size categories; and (iii) total biomass in trophic levels and taxonomic groups. Patterns of biomass and population abundance with body size and trophic level have been studied both within [7,25] and among [8,26–28] taxonomic groups at least since the time of Elton [2], in both aquatic [29–31] and terrestrial [9,10,32] systems, and have been increasingly important descriptors of community structure in recent years [9,10,33–36]. The patterns we use provide a reasonably comprehensive description of an ecological community. In testing differential equation models of community dynamics, we help answer the question of whether this widely used modelling framework actually is a sensible tool for understanding structure–dynamics links in ecosystems. If parameters are not found for which the models can reproduce the gross and commonly seen patterns described above, then model usefulness for any purpose will be questionable, whereas finding parameters that reproduce the patterns will support use of the models.

## 2. Methods

### (a) Community patterns that a model should reproduce

Empirical patterns that emerge in ecological communities by considering the average population densities (*N*) and body masses (*M*) of all species present have become widely studied [8–10,26,27,29,34,35,37]. The common power-law form of the *N*-versus-*M* relationship has broad implications for community- [9] and ecosystem-level [32] theories. The relationship indicates the average abundances of all species and hence provides a fairly comprehensive summary of the community. Species *N*-versus-*M* patterns are strongly affected by trophic structure, because consumer population densities are supported by biomass and nutrients that flow through trophic links [9,28,32]. Another commonly used form of mass–abundance relationship, this one ignoring species distinctions, is the classic abundance or size spectrum [30]. Log total *N* is computed in equally spaced log(*M*) bins and plotted against (log-scale) bin centres, with approximately linear results commonly occurring. Linear regressions fitted to species-specific or binned log(*N*)-versus-log(*M*) data provide a simple description of major patterns in the average abundances of species and body mass categories, respectively. Prior work showed that both measures can vary systematically among ecosystems [8–10,34].

**Figure 1.**
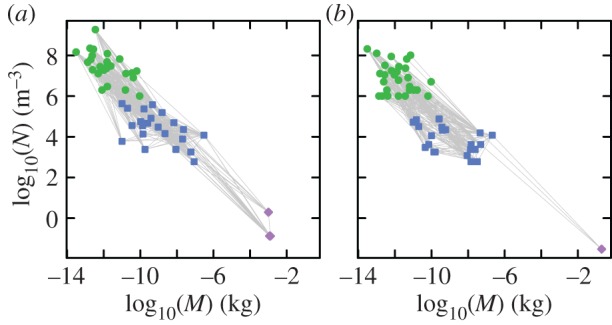
The communities of Tuesday Lake sampled in (*a*) 1984 and (*b*) 1986. Phytoplankton are shown by green circles, invertebrates by blue squares and fish by purple diamonds. Light grey lines indicate trophic links. Communities include 50 species and 269 trophic links in 1984, and 51 species and 241 links in 1986. Taxa are highly resolved, with 48 of the 50 food web nodes in 1984 and 49 of the 51 in 1986 being species and the remaining two taxa in both webs resolved either to genus level or described as unclassified flagellates.

The above patterns were quantified [26,27] for the pelagic epilimnion community of Tuesday Lake (figure 1 for the species-specific pattern), which was sampled during summer stratification in 1984 and 1986 [31]. Species lists, trophic links and species average *M* and *N* were quantified for Tuesday Lake in both years; these data are available [27,38]. Only about half of the species from 1984 were detected in 1986, probably largely owing to a lake-scale experimental manipulation performed in 1985; however, many structural features of the two communities are very similar [27]. Dynamic data at the functional-group level of taxonomic resolution have been collected for Tuesday Lake [13], but we did not use these data, because we do not seek to model the detailed dynamics of functional groups in the lake, but rather to assess the general realism of a modelling framework at the species level of resolution, by examining whether it can reproduce community patterns found in Tuesday Lake and many other systems. Dynamic data of this kind are also extremely rare, even at the functional-group level, whereas the data we use are more common.

### (b) The model

The model represents each population by a stock of biomass and models changes in biomass, *B_j_*, of the *j*th species as2.1
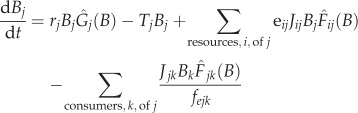
[11,39]. The parameters *r_j_*, *J_ij_* and *T_j_* were computed using allometric relationships that assume negative quarter power scaling [11,39]. The parameter *r_j_* is the mass-specific growth rate of the *j*th producer, 
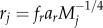
, where *a_r_* is derived from empirical data of species' maximum growth rates and body masses, *f_r_* is the fraction of maximum rate realized in a given ecological context and *r_j_* is zero for non-producers. The parameter *J_ij_* is the mass-specific ingestion rate of the *j*th consumer, 
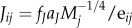
, for all *i* consumed by *j*. We used two empirically derived values of *a_J_*, one for invertebrates and one for vertebrate ectotherms, both derived from data on maximum ingestion rates. We also used two values of *f_J_*, the fraction of maximum ingestion rate that is realized, one for invertebrates and one for vertebrate ectotherms. The parameter *T_j_* is the mass-specific respiration rate of the *j*th consumer, 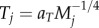, again with two values of *a_T_* used, one for invertebrates and one for vertebrate ectotherms; both were computed from ‘typical’ metabolic or respiration rate data. *T_j_* is zero for producers. Values of *a_r_*, *a_J_* and *a_T_* were previously determined [11] from empirical data, whereas *f_r_* and the two *f_J_* were unknown parameters.

The terms 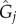 and 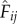 are the normalized (between 0 and 1) growth model and functional response, respectively, both of which are functions of the biomass densities of all populations (*B*). We used functional forms that had been used with our dynamical model in previous studies [20,22,23,37,40]. The growth model is2.2
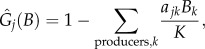
in which producers compete for a global carrying capacity, *K*. We set all competition coefficients, *a_jk_*, equal to 1, for the sake of simplicity. The functional response is2.3
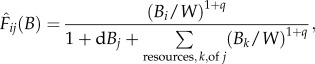
where *q* = 0 produces a type II response and *q* > 0 produces a type III sigmoid response. A type III response models switching between resource species, i.e. the consumer's apparent preference for resources depends on the relative densities of its resource species [39]. The higher the value of *q*, the closer the functional response is to a step function. The parameter *W* is the half-saturation biomass: the biomass density at which the functional response results in a value of 0.5. The parameter *d* controls the amount of intraspecific predator interference. Values of *d* > 0 reduce consumption rates of the *j*th population as the *j*th population becomes more common [22,37]. Not all consumers ingest everything that they kill, represented by the ingestion efficiency, *f_eij_*, which characterizes the amount of biomass removed from *i* that is ingested by *j*. The assimilation efficiency *e_ij_* is the fraction of biomass of *i* ingested by *j* that is actually converted to biomass of *j*. The model parallels models used in earlier works [11,20,22,39]. Time in the final model equations was normalized to the growth rate of the primary producer with the smallest body mass; model details are presented in the electronic supplementary material.

Running a simulation of the model required the set of trophic links for the community as well as the body mass, initial biomass density and metabolic category (either producer, invertebrate or vertebrate ectotherm) for each population. All of this information was taken from the community being simulated.

### (c) Simulations and model–data agreement

Simulations were started at the empirical, measured biomass densities (*B*_data_, kg m^–3^), computed from the product of the measured population densities (*N*_data_, individuals m^–3^) and body masses (*M*, kg). All simulations were run to stationary state, and the resulting densities, *B*_sim_, were used to compute the stationary-state population densities *N*_sim_ = *B*_sim_/*M*. Simulations were terminated when every population's biomass density reached either an equilibrium or stable oscillations; in the latter case, final biomass densities *B*_sim_ were time averages. Details on detecting extinctions and stationary states and our conditions for terminating simulations are in the electronic supplementary material.

Our measures of model–data agreement compare *N*_sim_ with *N*_data_ for all species, beginning by computing the number of species persisting. Given a community of *s* species,2.4
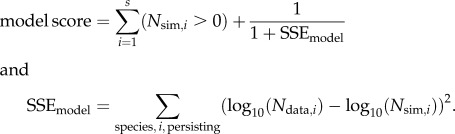
The second term in the model score formula varies between 0 and 1, so that the model score has a maximum value of 1 plus the number of species persisting. The degree of model–data agreement according to these scores assesses the extent to which major patterns in mass–abundance relationships can be explained as the dynamical outcome of the model.

For simulations in which all species persisted, we compared the sum-squared error (SEE), SSE_model_, with the equivalent SSE measure for an ordinary linear regression, here called SSE_regression_, in order to measure whether the dynamical model or ordinary linear regression was a better description of data. After performing an ordinary linear regression through a log_10_(*N*_data_)-versus-log_10_(*M*) scatter plot, SSE_regression_ was computed as the sum of squares of the residuals. Because this procedure is analogous to how SSE_model_ was computed, but replacing model predictions, log_10_(*N*_sim,*i*_), with regression-based predictions, SSE_model_ and SSE_regression_ are directly comparable.

### (d) Parameter space and optimizations

Seven parameters could not be determined from physiological allometries (*f_r_*, *f_J_* for invertebrates and vertebrate ectotherms, *d*, *q*, *W* and *K*; see the electronic supplementary material). We searched the space of undetermined parameters for good model–data agreement for the communities of 1984, 1986 and simultaneously for both years, each step through parameter space in the search requiring simulation of whole-community dynamics. The search is for model parameters that conform to physiological allometries and reproduce community patterns. The limits of sampling space and values of fixed parameters are in electronic supplementary material, table S1.

Each set of optimizations contained 1000 independent optimization runs starting from points sampled according to a Sobol low-discrepancy sequence (sobol.design in the pomp R package, v. 0.35-1 [41]). Optimizations were performed using the constrOptim function in R, with the Nelder–Mead method; the optimizer box-constrained parameter values to be within the sampling space. The two sets of optimizations that fitted to the 1984 or 1986 data used an objective function that ran a single dynamical model simulation to completion and returned the resulting model score. The objective function for the optimizations that fitted jointly to both communities ran a simulation of each community and returned the mean model score.

### (e) Randomizations

The optimizations described above will show whether parametrization by physiological allometries enables models to reproduce important community patterns. To further illuminate the importance of physiological allometries, it is necessary to check whether models with parameters not following physiological allometries are unable to reproduce community patterns. We randomly shuffled the parameters *r_j_* within metabolic categories. This destroys the body mass dependence of these parameters while retaining their aggregate statistical properties. In separate analyses, we randomly shuffled the *T_j_*, and, separately, the *J_ij_*, again within metabolic categories. We performed three separate randomizations for each of *r_j_*, *T_j_* and *J_ij_*, to prevent results from being overly dependent on the individual randomizations used. For each randomization, we repeated the optimization procedure outlined above, to see whether, or not, model agreement with community patterns could be obtained in spite of parameters not following physiological allometries. Optimizations using randomized parameters were carried out using 1984 data only.

All simulations were conducted using the R and C programming languages on the Imperial College High Performance Computing Cluster (R v. 2.11.1 [42]). Model differential equations were solved using the lsoda function in the R package deSolve v. 1.8.1 [43]. The Tuesday Lake communities both contained six producers with no consumers [27], which were removed for simulations.

## 3. Results

### (a) Model–data agreement

We found many sets of parameters that gave coexistence of all species, in both years (details in the electronic supplementary material, table S2). This result is notable in light of earlier results illustrating that the overwhelming majority of parameters for models of complex communities give model instability and/or species extinctions [4,5,20,21]. Although the region of parameter space is small for which species coexist as they do in nature, that region can easily be found if the search for it is guided by physiological allometries.

**Figure 2.**
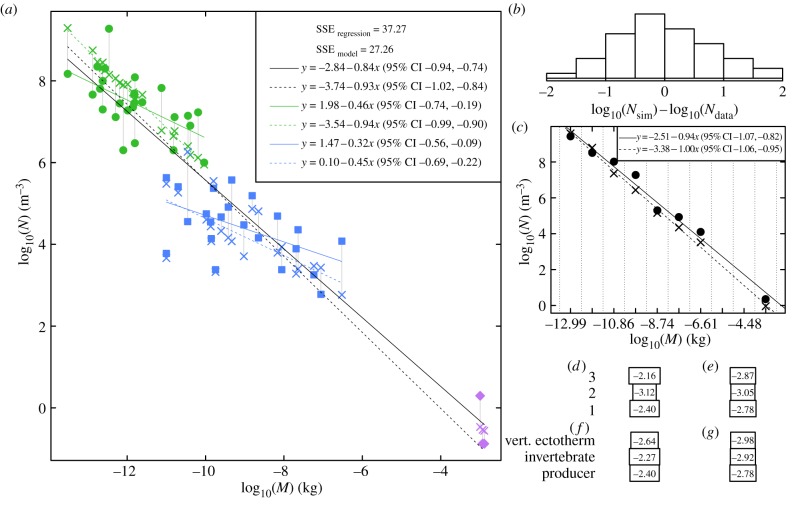
The performance of the best set of parameters fitted to the 1984 community. (*a*) Grey lines connect *N*_data_ (symbols and colours as in figure 1) and *N*_sim_ (crosses). Linear regressions through *N*_data_ (solid lines) and *N*_sim_ (dashed lines) are shown for producers, invertebrates and all populations. Regression equations with 95% confidence intervals of slope are shown in the legend. (*b*) Histogram of model–data residuals. (*c*) Abundance spectra for log_10_(*M*)-binned *N*_data_ (circles and solid line) and *N*_sim_ (crosses and dashed line). (*d*,*e*) *B*_data_ and *B*_sim_ binned by ‘prey-averaged’ trophic level [44], rounded down to the nearest integer. (*f*,*g*) *B*_data_ and *B*_sim_ binned by metabolic category.

With the best sets of parameters, the model not only achieved species persistence, but also successfully reproduced a variety of commonly observed community patterns (figure 2). The performance of the best set of parameters for the 1984 community produced SSE_model_ = 27.26, beating SSE_regression_ = 37.27 and showing that the dynamical model can predict numerical abundances in the community of 1984 better than a linear regression. Linear regression is a purely phenomenological model in this context, whereas our model is mechanistic, so the fact that our model does as well as regression is notable, even though it has more free parameters. The mass–abundance relationships through *N*_data_ and *N*_sim_, as given by linear regression through log-transformed data, were remarkably similar both for the whole community and within the phytoplankton and zooplankton subgroups; we did not examine this relationship for the three fish species as they have very similar body masses. The abundance spectrum of *N*_sim_ was also very close to that of the measured data. Total simulated and real biomasses were within an order of magnitude: 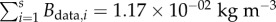; 
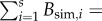
 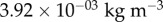. Biomass pyramids for *B*_data_ and *B*_sim_, split by trophic level and also by metabolic category, were very similar. A histogram showing the distribution of the residuals log_10_(*N*_sim_) − log_10_(*N*_data_) across species indicates that model errors were approximately normally distributed with mean close to zero (figure 2). Similar assessments using 1986 data and using data from both years jointly (see electronic supplementary material figure S1) show that species coexistence was achieved, and the model also performed reasonably well in those cases, although with patterns substantially less well matched than for 1984. Electronic supplementary material figure S2 plots biomass against time for the best parameters, showing that all populations reached equilibrium.

### (b) Parameter constraints

**Figure 3.**
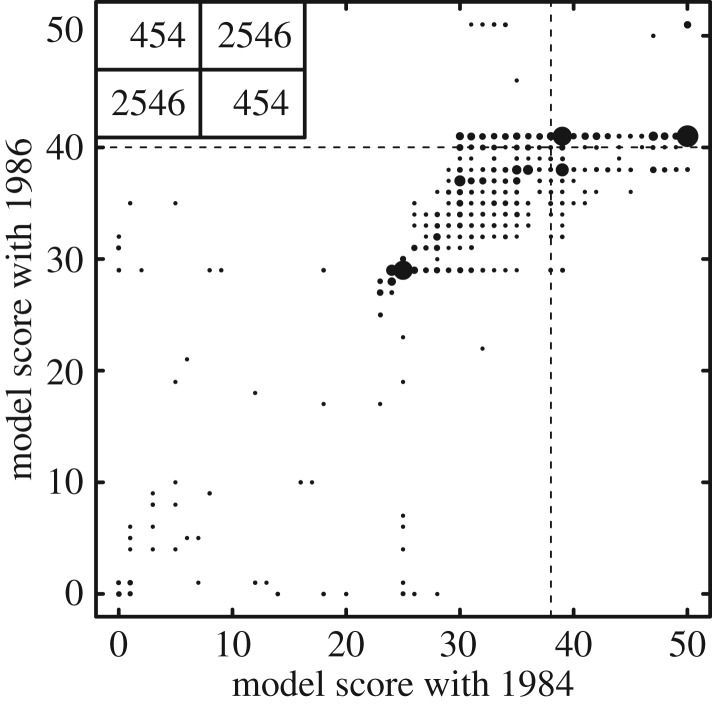
Model score for 1986 versus model score for 1984 for 6000 points in parameter space. The graph shows model score values rounded to the nearest integer. Diameters of circles are proportional to the number of model score values at that location. Dotted lines mark the median un-rounded model score values and the box shows the number of un-rounded values in each quadrant.

All three fittings (1984, 1986 and both years jointly) resulted in similar values of the seven fitted model parameters (see electronic supplementary material, figure S3), showing that constraints imposed by the requirement that models reproduce community patterns are broadly consistent for different communities of the same type. We here take the communities of 1984 and 1986 to be of the same type because they come from the same lake, but to represent different communities because of the manipulation in 1985 and because species composition changed by about half from 1984 to 1986. In both years, there was a clear lower bound to the growth rate of producers (*f_r_*), below which producers do not supply enough biomass for consumers to persist. There were also lower bounds to rates of ingestion (*f_J_*) for both invertebrates and vertebrate ectotherms. Coexistence required a minimum global carrying capacity, *K*, in both years. The value of the predator interference parameter, *d*, seemed unimportant to model performance in 1984, but high values were required for good performance when compared with 1986 data. All optimization endpoints with species coexistence had values of *q* greater than zero, corresponding to a type III functional response. The lowest value of *q* across all endpoints was 0.34, showing that coexistence required only a relatively weak type III response [22]. To further validate the commonality of parameter constraints imposed by community patterns in 1984 and 1986, we examined how model scores in the 2 years were correlated. Each of our three sets of fitting contained 1000 start points and produced 1000 endpoints. For each of these 6000 points in parameter space, we plotted the model score values for the 1986 community against those for 1984 (figure 3). Association was clear, with Pearson correlation coefficient 0.803. Electronic supplementary material, figure S4 shows the relationships among the seven parameters for each model fitting exercise.

The region in parameter space of ‘good’ model parameters was larger for 1984 than for 1986. The performance of the model for the 3000 optimization endpoints is summarized in the electronic supplementary material table S2. Parameters that fit well to the 1984 data, when applied to the 1986 data, did not produce good agreement. Most parameters that were obtained via the 1986 optimization also worked well for 1984 without modification. The number of simulations for each optimization set is presented in the electronic supplementary material, table S3.

### (c) Necessity of parameter allometries

Optimizations using randomized parameters never resulted in model scores as high as those using parameters that followed physiological allometries. For two of the three randomizations of the *r_j_* parameters, no optimizations even obtained coexistence of all species. For the third randomization, coexistence was achieved, but with a minimal SSE_model_ for simulations with coexistence of 58.41, higher than the value SSE_model_ = 27.26 obtained using allometries. For one of the three randomizations of the *T_j_* coexistence was never achieved, and for the other two randomizations, minimal SSE_model_ across simulations showing coexistence was 28.95, again higher than 27.26. None of the three randomizations of the *J_ij_* ever led to coexistence in any simulation. These results not only indicate the importance of allometric parametrizations, but also suggest an ordering of importance: allometry in ingestion rates, *J_ij_*, is most important for model–data agreement, and allometry in the *T_j_* is least important.

## 4. Discussion

Results provide an important message: that allometrically parametrized differential-equation models of community dynamics can recreate, with reasonably good accuracy, important large-scale quantitative patterns commonly seen in data. Had parameters not been found for which the models could reproduce the patterns examined here, model usefulness for any purpose would have been questionable. We found the reverse. Our results buttress the growing use of allometrically parametrized models [11,20,21,23] as reasonable tools for building understanding of community dynamics and the relationship between structure and dynamics.

In addition, our randomization results show that not only are physiological allometries sufficient to reproduce community patterns, they are also necessary: models with parameters not respecting physiological allometries cannot reproduce the patterns. The patterns are widespread and important. Therefore, our results show that research using models parametrized without respect to physiological allometries may be ecologically irrelevant.

### (a) Comparison with prior work

It had previously been shown that equation (2.1), when parametrized using body mass data, has qualitatively realistic properties, including stability [20]. However, it seems probable that many dynamical mechanisms confer stability and other qualitative features also found in real systems, and not all such mechanisms are ecologically important—stability *per se* is not a sufficiently exacting test of a model. Our work builds on earlier work by showing not only that allometrically parametrized models agree with observation qualitatively, but also quantitatively, in terms of log(*N*)-versus-log(*M*) scaling and good agreement with precise *N* values. This is a much more exacting model test and is more indicative that models contain correct mechanisms. Our result that allometric parametrization is not only sufficient but also necessary to reproduce important patterns also extends prior work and indicates that allometric parametrization is the way forward for studies of community dynamics.

Boit *et al.* [24] also examined the question of whether allometrically parametrized models can reproduce quantitative empirical patterns; to the best of our knowledge, theirs is the only prior study to do so. Our results complement those of Boit *et al.* in two related ways, and avenues of future research proposed below are informed by both studies. First, Boit *et al.* primarily examined whether their models could reproduce seasonal dynamical patterns, whereas we examined common average patterns of population density. Both seasonal fluctuations and average population density regularities should be reproduced by a good modelling framework, so our results combine with those of Boit *et al.* to increase support for models. The models of Boit *et al.* were driven by observed fish mortality, whereas our models were not externally forced and Tuesday Lake was not fished prior to 1984 [27]. Second, the dataset of Boit *et al.* was of low taxonomic resolution but of high temporal resolution and extent, whereas ours was of high taxonomic resolution. Multi-annual whole-community time series such as those of Boit *et al.* are extremely rare and difficult to obtain, whereas our data can be gathered in one summer. One goal of modelling community dynamics is to predict the consequences of perturbations. For practical use, prediction should be based on data that can be gathered in reasonable time.

### (b) Future research

Comparisons of model–data agreement results for 1984 versus 1986 and for phytoplankton versus zooplankton versus the whole community provide important lessons, discussed below, about how model–data agreement might be improved for Tuesday Lake. More importantly, and also discussed below, these comparisons indicate how a formal statistical approach based on the conceptual framework provided here might be constructed for model selection among multiple models to determine what mechanisms are important for community dynamics and what models should be used for forecasting. This approach is the way forward we recommend for increasing the realism of models of Tuesday Lake and other systems.

Model–data agreement was worse for 1986 than for 1984, and examining this difference indicates ways in which models of Tuesday Lake might be improved in future work. We considered four possible improvements and fitted models for two of them. First, the largemouth bass, *Micropterus salmoides*, present in 1986 but not in 1984, is known to consume non-pelagic species [31,45,46]. External subsidies to bass in 1986 may help explain why model–data agreement was less good for 1986 than 1984. Second, bass are atypical in that they were artificially introduced to the lake, and other species found in the lake in 1986 were much smaller than typical adult bass prey. The only prey items in Tuesday Lake 1986 in the preferred prey size range of adult bass were young bass. Consumption of young bass by adult bass may therefore have been accentuated in Tuesday Lake in 1986 relative to cannibalism rates that occur when other prey are available, and this may have effectively reduced the efficiency of trophic transfer from non-bass prey species to bass. Atypical respiration rates of bass may also help explain why model–data agreement was less good for 1986 than 1984. Third, poor model–data agreement may have come about because Tuesday Lake had not reached a new steady state by 1986 following the perturbation imposed in 1985, whereas model–data comparisons used model steady states. Fourth, species-specific deviations from rate allometries in other species besides bass could have cause model–data discrepancies. We fitted models to 1986 data to test the first two hypotheses. Best results had all species persisting with SSE_model_ equal to 135.10 for the external subsidy hypothesis, and 99.00 for the atypical bass efficiency and respiration hypothesis, compared with 135.23 for the unmodified model. These appear to be improvements, but our approach does not allow judgements on whether these values are statistically significantly better given that they require additional free parameters. Models were not fitted for the latter two hypotheses, because a large number of additional parameters would have been needed, and spurious ‘improvements’ in model fit could easily have been observed. Boit *et al.* also made comparisons among models of their system, although their approach, like ours, is also not a formal statistical approach and also does not allow for judgements as to whether apparent improvements are significant. Although some models of Boit *et al.* appeared substantially better than others, some of their models apparently also had as many as 55 additional free parameters, and a variety of adjustments were made by hand that seem likely to contribute additional degrees of freedom.

Model–data agreement in this study was better for zooplankton and for all species together than it was for phytoplankton: slopes of log(*N*)-versus-log(*M*) regressions through model output for 1984 were within 95% CIs of the slopes of regressions through data for zooplankton and for all species, but not for phytoplankton (figure 2*a*). This suggests one more possible improvement to the model that could be considered in future work, to its phytoplankton competition component. Currently, all phytoplankton compete equally and are subject to an overall biomass carrying capacity. In fact, phytoplankton compete for nutrients and light, and recent literature explores how this competition depends on body size [47]. Encorporating a more realistic competition model may improve overall fit. This proposal was not carried out because of the statistical considerations mentioned above and discussed in detail below, but will probably be a fruitful avenue of research once statistics are improved.

Our study and Boit *et al.* did not develop methods that allow formal statistical comparison of different models because developing such methods will be a major challenge, and would have been premature before having evidence that models can at least approximately reproduce quantitative patterns. Because our work and that of Boit *et al.* has demonstrated they can, we describe two characteristics a future formal approach should have. First, the approach should be based on statistical likelihood, so model selection tools such as the Akaike information criterion can be used to weight alternative models while accounting for both quality of fit and model complexity. Population ecologists have used likelihood and model selection with great success to infer mechanisms that drive the dynamics of important population systems such as cholera [48], *Tribolium* beetles [49,50] and others [51]. The approach is also used in other fields [52,53], and is increasingly seen as the future for models and data in ecology [54,55]. But, it has not been applied to whole-community dynamics. Likelihood and model selection in population ecology exploit high-quality time-series datasets, almost never available for communities. So community-level data previously appeared too scarce for the methods but our study suggests that if existing community data are combined with literature metastudy data on body size and taxonomic correlates of model parameters, fitting can be successful. A key insight from our results is that community data are in fact not scarce, they are just more heterogeneous than the time series of population ecology: relevant data encompass not just measurements from the community but also literature data constraining physiological rates. So the second feature of our proposed approach is that it should unify community data with literature data on body size and taxonomic correlates of rates within a single likelihood framework. The same concepts could be applied to community matrix, autoregressive and Markov chain approaches to studying community dynamics, whenever it is possible to constrain parameters using physiological allometries.
